# Global perspectives on dietary management of small intestinal bacterial overgrowth (SIBO): a systematic review of clinical guidelines, consensus statements and expert recommendations

**DOI:** 10.3389/fmed.2026.1952995

**Published:** 2026-09-28

**Authors:** Magdalena Jodkiewicz, Justyna Malinowska, Karolina Marek-Woźny

**Affiliations:** Medical Center of Dietetics and Health Education, National Institute of Public Health NIH - National Research Institute, Warsaw, Poland

**Keywords:** antibiotic therapy, dietary fiber, dietary therapy, gut microbiota, low-FODMAP diet, probiotics, SIBO

## Abstract

**Introduction:**

Small intestinal bacterial overgrowth (SIBO) is a disorder associated with gastrointestinal symptoms, alterations in gut microbiota composition, and a high rate of recurrence. Although antibiotic therapy remains the cornerstone of treatment, dietary interventions are increasingly used as adjunctive strategies to improve symptom control and support long-term management. However, international recommendations regarding dietary management remain heterogeneous and lack standardization. Therefore, the aim of this systematic review was to analyze and compare dietary recommendations for the treatment of SIBO presented by international scientific organizations and recent expert reviews.

**Methods:**

This guidelines review was conducted in accordance with PRISMA 2020 recommendations. PubMed and Cochrane Library were searched for publications published between January 2016 and January 2026. Eligible documents included clinical guidelines, consensus statements, official position papers, and supplementary expert review articles addressing dietary management in SIBO. Data were extracted on the role of dietary therapy, low-FODMAP recommendations, dietary fiber, probiotics, prebiotics, treatment priorities, and the reported impact of dietary interventions on the gut microbiota. A qualitative synthesis of recommendations from different geographic regions was performed.

**Results:**

Five key documents from North America, South America, Europe, and the Asia-Pacific region met the inclusion criteria. Dietary therapy was consistently regarded as supportive rather than curative, although its perceived importance varied across regions. The low-FODMAP diet was the most frequently discussed dietary intervention and was generally recommended for symptom relief, particularly for bloating, abdominal pain, and altered bowel habits. Nevertheless, evidence supporting its role in eradicating SIBO remains insufficient. Recommendations regarding dietary fiber, probiotics, and prebiotics were heterogeneous and largely emphasized individualized use. Antibiotic therapy and management of underlying predisposing factors remained the central therapeutic strategy in all analyzed regions.

**Conclusion:**

Despite increasing interest in nutritional approaches, substantial international variability exists in dietary recommendations for SIBO. Current evidence supports dietary therapy primarily as an adjunct to medical treatment and a tool for symptom management. High-quality prospective studies are needed to establish standardized, evidence-based dietary recommendations and to clarify the long-term effects of dietary interventions on the gut microbiota and disease recurrence.

## 1 Introduction

Small intestinal bacterial overgrowth (SIBO) is a disorder characterized by an in-creased number of bacteria in the small intestine or the presence of microorganisms that do not normally occur in this section of the gastrointestinal tract. These changes result in gastrointestinal symptoms, primarily including bloating, abdominal pain, excessive gas, diarrhea, constipation, and malabsorption of nutrients. Disorders of the gut microbiota, including SIBO, are considered one of the potential pathophysiological mechanisms underlying IBS, particularly in its diarrhea-predominant form (1). SIBO is diagnosed in 33.8% of individuals reporting gastrointestinal symptoms who have undergone a breath test. This condition is significantly correlated with cigarette smoking, bloating, abdominal pain, and anemia. The use of proton pump inhibitors is considered an important factor contributing to the development of SIBO. Furthermore, the likelihood of its occurrence increases with age but is not associated with gender or ethnicity (2). Recurrence after treatment is common and has been reported in a substantial proportion of patients, highlighting the need for long-term management strategies, including nutritional interventions (3).

Despite growing interest in SIBO, significant controversy remains regarding both the diagnosis and treatment of this condition. Most current guidelines focus on the use of antibiotics, particularly rifaximin, while the role of dietary interventions remains less clear. In clinical practice, elimination diets are increasingly being used, particularly a diet low in fermentable oligo-, di-, and monosaccharides and polyols (low FODMAP); however, evidence supporting its effectiveness in treating SIBO is limited (4).

A diet low in fermentable oligosaccharides, disaccharides, monosaccharides, and polyols (low FODMAP) is a relatively new therapeutic approach. The first study, published in 2008, was a double-blind, randomized, placebo-controlled trial that involved the re-introduction of fructans and fructose in patients with irritable bowel syndrome (IBS) who had previously responded well to a low-FODMAP diet (5). The dietary approach consists of three stages, beginning with an elimination phase in which all foods containing these compounds are excluded. If there is a marked improvement in symptoms after approximately 6–8 weeks, the next groups of FODMAPs are gradually reintroduced in the second stage. This makes it possible to identify which ingredients trigger symptoms, allowing for the development of a personalized, long-term eating plan that effectively alleviates IBS symptoms as part of the third phase of the diet (6, 7).

Managing SIBO remains a significant clinical challenge due to high recurrence rates and the limitations of currently available therapeutic approaches. While antibiotic therapy remains the cornerstone of bacterial eradication, dietary interventions are increasingly used as supportive strategies for symptom management. However, international clinical guidelines, consensus statements, and expert reviews provide heterogeneous dietary recommendations, ranging from fermentable carbohydrate restriction to individualized use of fiber, probiotics, and prebiotics. This lack of standardization complicates clinical decision-making and highlights the need for a comprehensive synthesis of the available evidence. Therefore, the aim of this study was to analyze and compare dietary recommendations for the treatment of SIBO presented by scientific organizations and recent expert reviews.

## 2 Materials and methods

### 2.1 Study design

A systematic review was conducted on dietary recommendations used in the treatment of SIBO in accordance with the PRISMA 2020 statement. The review aimed to systematically identify and compare international recommendations regarding dietary management of small intestinal bacterial overgrowth (SIBO). The review protocol was developed *a priori*; however, it was not registered in PROSPERO.

### 2.2 Research question (PICOS)

The review was designed to address the following research question:

How do international clinical guidelines, consensus statements, and official position papers differ in their recommendations regarding dietary management of small intestinal bacterial overgrowth (SIBO)?

The research question was formulated according to the PICOS framework:

- Population (P): adults diagnosed with small intestinal bacterial overgrowth (SIBO) or patients with SIBO-related gastrointestinal symptoms.
- Intervention (I): dietary interventions used in the management of SIBO, including low-FODMAP diets, dietary fiber modification, probiotics, prebiotics, elemental diets, and other nutrition-based strategies.
- Comparator (C): standard medical care, antibiotic therapy, alternative dietary approaches, or differences in recommendations across geographical regions.
- Outcomes (O): recommendations regarding symptom management, SIBO eradication, prevention of recurrence, gut microbiota modulation, and overall treatment strategy.
- Study Design (S): clinical guidelines, consensus statements, and official position papers and, where no regional guidance documents were available, supplementary review articles addressing dietary management of SIBO.

The primary objective of this review was to compare recommendations regarding the role of dietary therapy in SIBO management across different world regions. Particular attention was given to recommendations concerning low-FODMAP diets, dietary fiber intake, probiotic and prebiotic supplementation, and the integration of nutritional therapy with antibiotic treatment. Additionally, the review evaluated how the impact of dietary interventions on the gut microbiota was interpreted in different international documents.

### 2.3 Search strategy

A literature search was conducted in PubMed and Cochrane Library in January 2026. Search terms included: (“small intestinal bacterial overgrowth” OR “SIBO”) AND (“diet” OR “low FODMAP” OR “nutrition”) AND (“guideline” OR “consensus” OR “recommendation”). Only publications published in English between January 2016 and January 2026 were considered. A total of 26 documents were found, of which 23 were excluded because they did not match the scope of our search. Additionally, two European review articles were identified through supplementary hand-searching and incorporated into the qualitative synthesis because no European clinical guidelines, consensus statements, or position papers addressing dietary management of SIBO were identified, resulting in a total of five included documents (Figure 1).

**Figure 1:**
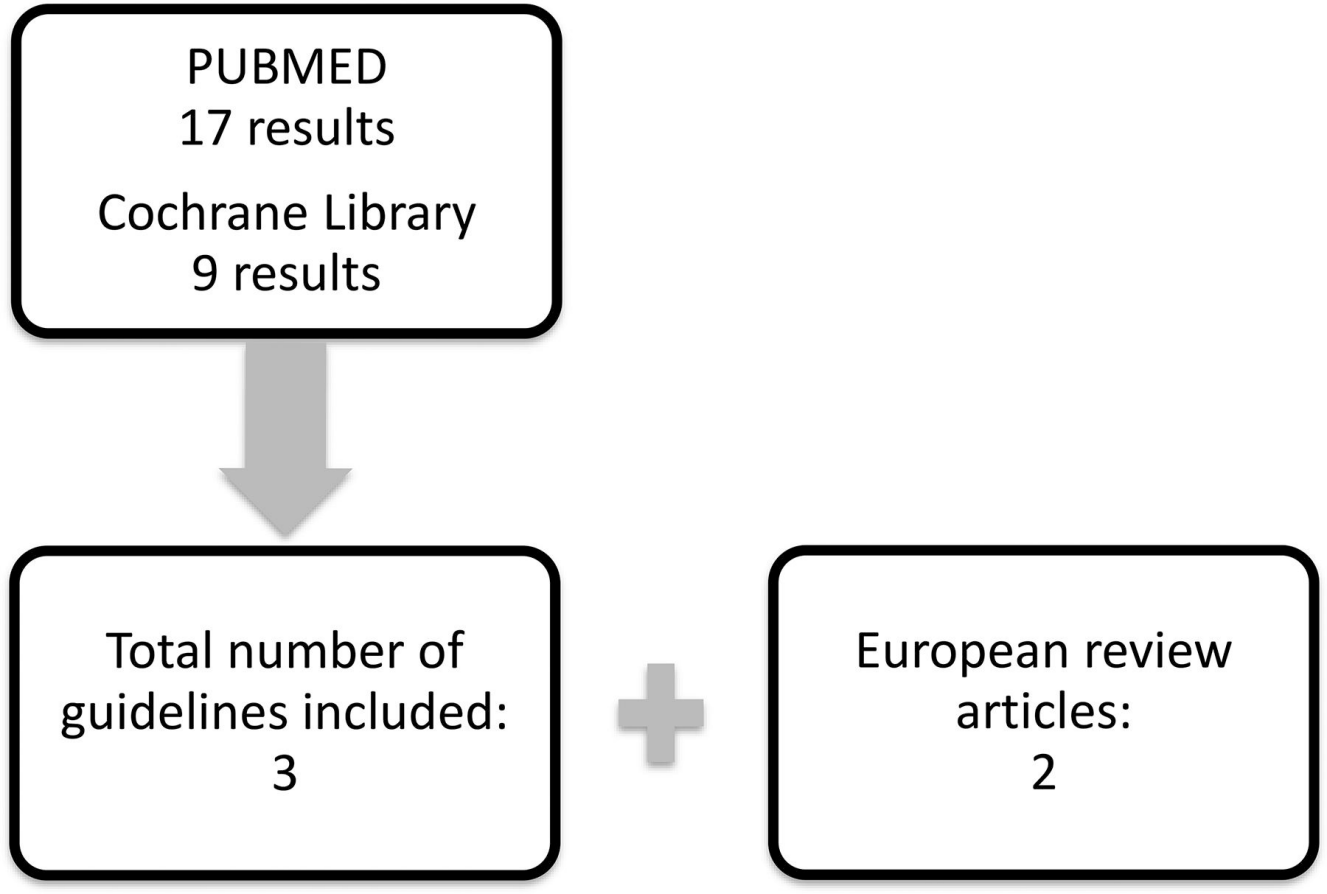
Flow diagram adapted from PRISMA 2020 guidelines, illustrating the identification of records via databases (PubMed and Cochrane Library, January 2026; *n* = 26) and manual hand-searching for regional European evidence (*n* = 2), culminating in a final qualitative synthesis of 5 documents.

### 2.4 Eligibility criteria

Documents were selected according to predefined eligibility criteria established before the literature search. The inclusion criteria were developed based on the objective of identifying documents that provide recommendations regarding dietary management of small intestinal bacterial overgrowth (SIBO).

Inclusion criteria:

- clinical guidelines,
- consensus statements,
- official position papers.

Exclusion criteria included case reports, conference abstracts, non-English publications, and studies not addressing dietary management of SIBO.

The following documents were included in the analysis: guidelines from the American College of Gastroenterology (ACG) (8), an Asia-Pacific expert consensus developed by the Indian Neurogastroenterology and Motility Association (9), a position statement from the Brazilian Federation of Gastroenterology (10), and due to the lack of European guidelines, two European review articles were additionally incorporated through supplementary hand-searching to provide representation of European perspectives on dietary management in SIBO (3, 4). Based on the available data, a qualitative comparison of recommendations from different regions of the world was conducted.

Extracted domains included the role of dietary therapy, low-FODMAP recommendations, dietary fiber, probiotics, prebiotics, treatment priorities, and reported effects on gut microbiota.

### 2.5 Assessment of methodological quality

Because the review focused primarily on clinical guidelines, consensus statements, position papers, and supplementary review articles rather than primary clinical studies, a formal risk-of-bias assessment designed for intervention studies was not considered applicable. Instead, methodological characteristics of the included documents, including transparency of development methods, evidence base, and reporting of recommendations, were evaluated qualitatively and taken into account during interpretation of the findings.

## 3 Findings

### 3.1 A comparative analysis of dietary recommendations for SIBO in different regions of the world

The analysis examined key elements of dietary management in SIBO, including the role of the low-FODMAP diet, the importance of dietary fiber, the use of probiotics and prebiotics, the role of dietary therapy versus antibiotic therapy, and the impact of dietary interventions on the gut microbiota. The findings suggest differences among the documents originating from different regions with respect to the strength of recommendations and the interpretation of available scientific evidence (Table 1). In addition, contemporary European literature highlights that dietary interventions are primarily considered supportive strategies aimed at symptom control, whereas antibiotic therapy and correction of underlying predisposing factors remain the main therapeutic approaches in SIBO management (3).

**Table 1:** A comparison of dietary recommendations for SIBO in different regions of the world.

Parameter	ACG (USA)	Brazilian position paper	Asia-Pacific consensus	Ahmed et al. (UK)	Wielgosz-Grochowska et al. (Poland)
Role of dietary therapy	Supportive role; evidence insufficient to recommend diet as primary treatment	Important adjunct within a multimodal treatment strategy	Potentially beneficial, but evidence remains limited	Supportive role; symptom-oriented management	Potentially important adjunctive therapy, especially in patients with overlapping IBS
Low-FODMAP diet	No formal recommendation due to limited evidence in SIBO	Recommended as an adjunctive option for symptom relief	No consensus recommendation	May improve symptoms, but evidence is insufficient for routine use	Most extensively discussed dietary approach; may reduce bloating, abdominal pain and gas production
FODMAP restriction	Not specifically addressed	Considered part of symptom-control strategies	Individualized according to symptoms and clinical context	May reduce bacterial fermentation substrates	Mechanistically justified to decrease fermentation and symptom burden
Dietary fiber	No clear recommendations	Supportive role depending on clinical presentation	No specific recommendation	Excess fermentable fiber may aggravate symptoms in some patients	Individualized approach; tolerance depends on fiber type and symptom profile
Probiotics	Limited and inconsistent evidence; not routinely recommended	May be used as adjunctive therapy	Evidence remains inconclusive	Evidence inconsistent; routine use not recommended	Potential benefit suggested, but current evidence remains heterogeneous
Prebiotics	No routine recommendation	Possible supportive role with individualized use	Not established	May worsen symptoms due to increased fermentation; insufficient evidence	Potential microbiota benefits but may aggravate symptoms during active SIBO
Treatment cornerstone	Antibiotic therapy and treatment of underlying causes	Combination therapy including antibiotics and management of predisposing factors	No single standardized approach; management individualized	Antibiotics and correction of predisposing conditions	Combined medical and nutritional management
Impact of diet on the microbiota	Secondary consideration; insufficient evidence for long-term effects in SIBO	Clinically acknowledged but poorly characterized	Insufficiently understood	Long-term dietary restriction may reduce microbial diversity	Low-FODMAP diet may reduce commensal bacterial populations; reintroduction phase is essential

ACG, American College of Gastroenterology; FODMAP, fermentable oligosaccharides, disaccharides, monosaccharides and polyols; IBS, irritable bowel syndrome; SCFA, short-chain fatty acid; SIBO, small intestinal bacterial overgrowth.

### 3.2 The role of the low-FODMAP diet

A diet low in fermentable oligo-, di-, and monosaccharides and polyols (low-FODMAP) is the most widely studied dietary approach in the context of SIBO. In Europe and North America, this diet is viewed primarily as a tool for symptom relief rather than a causal treatment. It has been shown to effectively reduce bloating, abdominal pain, and irregular bowel movements; however, its effect on the eradication of SIBO remains unconfirmed (4). Similarly, Ahmed et al. emphasized that although low-FODMAP and other carbohydrate-restrictive dietary approaches may reduce symptoms associated with bacterial fermentation, current evidence is insufficient to recommend these diets as primary treatment modalities for SIBO (3).

In South America, according to the consensus of the Brazilian Federation of Gastroenterology, the low-FODMAP diet may be used as an adjunct to antibiotic therapy (10). At the same time, the literature highlights the potential limitations of this strategy. Long-term low-FODMAP dietary restriction has been reported to affect gut microbiota composition, particularly through reductions in Bifidobacteria, although findings regarding overall microbial diversity and short-chain fatty acid production remain inconsistent (4).

### 3.3 The importance of dietary fiber

The importance of dietary fiber in SIBO is not clearly established and depends on the type of fiber and the clinical context. Included publications discussed several fiber and prebiotic interventions, including psyllium, inulin, and partially hydrolyzed guar gum (PHGG). These products differ substantially in their fermentability, physiological properties, tolerability, and potential effects on gastrointestinal symptoms and gut microbiota. Some documents reported that selected fiber interventions may increase the abundance of SCFA-producing bacteria and may be associated with improved intestinal barrier function, while others emphasized the need for individualized use depending on symptom profile and tolerance (4). Among these interventions, PHGG deserves particular attention because direct evidence is available in patients with SIBO. A randomized clinical trial demonstrated higher breath-test normalization rates with rifaximin combined with PHGG compared with rifaximin alone; however, this finding originated from a relatively small study and requires confirmation in larger clinical trials. Some documents also reported that fiber supplementation may be associated with improvement in symptoms such as constipation, bloating, and abdominal pain. Excessive dietary fiber restriction was identified as a potential concern in some publications (4). Ahmed et al. additionally noted that excessive dietary restrictions may worsen nutritional status and potentially negatively affect the intestinal microbial ecosystem, highlighting the need for individualized dietary management (3).

### 3.4 Probiotics and prebiotics

The use of probiotics and prebiotics in the treatment of SIBO remains a subject of debate, and recommendations vary by region. According to Ahmed et al., evidence supporting routine probiotic supplementation remains inconsistent, and currently available studies do not allow for strong recommendations regarding their routine use in SIBO treatment (3). Reported effects appear to vary according to the specific microorganism, strain, formulation, and treatment protocol evaluated. Brazilian guidelines and selected clinical studies suggest a potential role for probiotics as adjunctive therapy, particularly in combination with antibiotic treatment (10, 11).

Prebiotics, although they potentially support the growth of commensal microbiota, may also exacerbate fermentation symptoms, which limits their use during the active phase of SIBO. For this reason, their use requires individualized management (3, 4).

### 3.5 The role of dietary therapy in relation to antibiotic therapy

In all regions analyzed, antibiotic therapy forms the basis of SIBO treatment. The American guidelines (ACG) emphasize that treatment should be primarily aimed at eradicating bacterial overgrowth, and that the use of antibiotics, particularly rifaximin, is the first-line strategy (8). The effectiveness of this form of treatment is con-firmed by clinical studies, which have demonstrated significant improvement in symptoms and high SIBO eradication rates depending on the antibiotic regimen used (12). This position is consistent with the review by Ahmed et al., who concluded that the primary therapeutic objective should be the reduction of bacterial overgrowth with antibiotic therapy and the correction of underlying predisposing factors, while dietary interventions should be considered supportive rather than curative measures (3). In Brazilian guidelines, a more integrated approach is observed, in which dietary therapy is part of combination therapy (10).

Comparative studies indicate that a low-FODMAP diet may be as effective as rifaximin in short-term symptom control; however, antibiotic therapy remains more effective in rapidly alleviating symptoms and is associated with higher patient adherence (13). Therefore, dietary therapy is most often considered as:

- a supplement to treatment,
- a component of long-term management,
- a strategy for preventing relapses.

The European literature largely supports this approach, emphasizing that dietary measures should be integrated with medical treatment rather than used as stand-alone interventions (3, 4).

### 3.6 The impact of dietary interventions on the gut microbiota

One of the key aspects distinguishing regional approaches is the interpretation of the diet's impact on the gut microbiota. European reviews reported that low-FODMAP dietary interventions may alter gut microbiota composition, with reductions in Bifidobacteria being among the most consistently reported findings, whereas effects on overall microbial diversity remain inconsistent (3, 4). Included publications suggested that dietary fiber and prebiotics may influence gut microbial composition and short-chain fatty acid production (4).

## 4 Discussion

This review demonstrated substantial international variability in dietary recommendations for SIBO despite broad agreement that dietary interventions should primarily be considered supportive rather than curative strategies. Across all analyzed regions, antibiotic therapy and management of predisposing factors remained the cornerstone of treatment, whereas recommendations regarding low-FODMAP diets, dietary fiber, probiotics, and prebiotics varied considerably. These findings highlight the absence of standardized global dietary guidance for SIBO and the limited evidence base supporting many nutritional interventions.

A comparative analysis revealed differences in approaches to dietary therapy for the treatment of small intestinal bacterial overgrowth (SIBO) among the documents originating from different regions of the world, which stem from both the limited quality of available scientific evidence and differing interpretations of the disorder’s pathophysiology.

The most pronounced differences concern the role of dietary therapy in the treatment regimen. In North America, in accordance with the guidelines of the American College of Gastroenterology, antibiotic therapy remains the mainstay of treatment, while dietary interventions are not considered first-line therapy and serve a supplementary role (8). The effectiveness of antibiotic therapy as the cornerstone of SIBO treatment is al-so confirmed by clinical studies, which have demonstrated significant improvement in symptoms and high rates of breath-test normalization and symptom improvement depending on the treatment regimen used (12). A similar pattern is observed in Europe, where dietary therapy focuses primarily on alleviating symptoms rather than eliminating the cause of the disease (4). Ahmed et al. emphasized that the primary therapeutic goal in SIBO is the reduction of bacterial overgrowth with antibiotic therapy and the identification and correction of predisposing factors, whereas dietary interventions should be regarded mainly as supportive measures aimed at symptom control (3).

In contrast to this approach, the consensus of the Brazilian Federation of Gastroenterology points to a more integrated treatment model, in which dietary interventions, including a low-FODMAP diet, constitute an essential component of supportive therapy used in conjunction with antibiotic therapy. This approach takes into account the multifactorial etiology of SIBO and the importance of modulating the gut environment, particularly in light of the high rate of disease recurrence (10).

The relationship between SIBO and irritable bowel syndrome (IBS) is widely debated, and disturbances in the gut microbiota represent one of the key pathophysiological mechanisms underlying these conditions (1). One of the key issues analyzed in this study was the role of the low-FODMAP diet. Although its effectiveness in reducing gastrointestinal symptoms is well documented, particularly in patients with irritable bowel syndrome (IBS), direct evidence supporting its effectiveness in patients with confirmed SIBO remains limited (4). Therefore, part of the rationale for dietary interventions in SIBO is extrapolated from studies conducted in IBS populations, which may not be fully representative of patients with confirmed bacterial overgrowth. This observation is consistent with the conclusions of Ahmed et al., who noted that despite the widespread use of low-fermentation and low-FODMAP dietary strategies in clinical practice, evidence supporting their effectiveness in eradicating bacterial overgrowth remains insufficient (3). Comparative studies have shown that this diet may provide symptomatic improvement similar to that of rifaximin in the short term; however, it is characterized by a slower onset of action and lower patient adherence (13). In a Polish study by Bogdanowska-Charkiewicz et al., based on a multivariate regression analysis, it was found that the use of antibiotic therapy (rifaximin, rifaximin with metronidazole, or rifaximin with neomycin) prior to starting the low-FODMAP diet increased the likelihood of a better response to the diet and increased the likelihood of a favorable response to the diet and improved well-being by approximately sevenfold. However, this finding should be interpreted with caution, as it was derived from a single observational study and does not necessarily translate into sustained long-term clinical remission or improved quality of life across broader SIBO populations (14). In contrast, a study by Gong et al. found that in a group of patients with SIBO-related halitosis that was resistant to antibiotic therapy, after following a low-FODMAP diet for 4 weeks, 80.85% of patients achieved breath-test normalization, and 78.72% showed an improvement in the concentration of dimethyl sulfide (the compound responsible for the unpleasant odor) in exhaled breath (15). While these findings are encouraging, breath-test normalization should not be interpreted as definitive evidence of SIBO eradication, and the clinical significance of this outcome requires further investigation. Although these results appear promising, they were obtained in a highly specific patient population with refractory halitosis and short-term follow-up. Therefore, their generalizability to the wider SIBO population remains uncertain (15). In contrast, the ESPGHAN Position Paper on the Use of the Low-FODMAP Diet in Pediatric Gastroenterology states that there is insufficient evidence to support the routine use of a low-FODMAP diet in the treatment of children with functional gastrointestinal disorders, including SIBO (16). It is worth noting, however, that children and adolescents are not included in the treatment recommendations for SIBO.

A significant concern is the potential impact of a low-FODMAP diet on the gut microbiota. Several studies have reported reductions in the abundance of Bifidobacteria during the restrictive phase of the low-FODMAP diet (3, 4). However, evidence regarding broader effects on microbial diversity, other bacterial taxa, and short-chain fatty acid production remains inconsistent. Furthermore, most available data relate to the initial restriction phase, whereas the complete low-FODMAP approach includes subsequent reintroduction and personalization phases that may modify or attenuate these microbiota-related effects. Consequently, the clinical relevance of these microbiota changes in patients with SIBO remains uncertain and requires further investigation.

It is also important to recognize that the impact of a low-FODMAP diet on the gut microbiota may depend on the overall quality and diversity of the diet. Even during FODMAP restriction, patients may consume a wide variety of plant foods, dietary fiber sources, and polyphenol-rich products. Therefore, microbiota-related outcomes may vary substantially depending on dietary implementation and adherence. Therefore, differences in dietary implementation may potentially influence microbiota-related outcomes, although this hypothesis requires further investigation (17).

The role of dietary fiber is equally complex. The literature suggests that soluble fiber may have a beneficial effect on the microbiota by increasing the production of short-chain fatty acids (SCFAs) and promoting the growth of anti-inflammatory bacteria (4). However, dietary fiber should not be considered a homogeneous intervention. Different fiber types vary substantially in their fermentability, gas-producing potential, and physiological effects. For example, psyllium, inulin, and partially hydrolyzed guar gum (PHGG) differ in their impact on gastrointestinal symptoms and microbial metabolism. Similarly, Ahmed et al. emphasized that nutritional interventions in SIBO should be patient-specific, as symptom severity, underlying predisposing conditions, and nutritional status may substantially influence dietary tolerance and clinical out-comes (3). Among the studied interventions, PHGG has generated particular interest because direct evidence is available in patients with SIBO. In a randomized clinical trial, supplementation with PHGG (5 g/day) in combination with rifaximin was associated with higher breath-test normalization rates compared with rifaximin alone. Although this finding originates from a relatively small study and requires confirmation in larger trials, it suggests that selected fiber-based interventions may have a potential adjunctive role in SIBO management (18).

The role of probiotics in SIBO remains uncertain. One of the challenges in interpreting the available evidence is that probiotics represent a heterogeneous group of interventions, with effects depending on the specific microorganism, strain, dose, formulation, and treatment duration. Consequently, findings from studies evaluating different probiotic preparations are not directly comparable. Current evidence suggests that selected probiotic formulations may provide benefit as adjunctive therapy; however, the overall quality of evidence remains limited and substantial heterogeneity exists across studies. In the randomized trial by Redondo-Cuevas et al., a multimodal intervention combining dietary modification, antibiotics, probiotics, and bioactive compounds was associated with clinical improvement (11). However, because multiple interventions were administered simultaneously, the independent contribution of the probiotic component cannot be determined. Particular interest has been directed toward Saccharomyces boulardii, which, as a probiotic yeast, is not directly affected by antibacterial antibiotics and may therefore be suitable for concomitant administration during antibiotic treatment. Nevertheless, evidence regarding its specific effectiveness in SIBO remains limited, and further well-designed studies are required before firm recommendations can be made. In a study by Bogdanowska-Charkiewicz et al., probiotic supplementation during a low-FODMAP diet was not associated with improved intervention outcomes (14). This finding further illustrates the heterogeneity of currently available evidence and the difficulty in drawing generalized conclusions regarding probiotic use in SIBO. Prebiotics, due to their potential to increase fermentation and exacerbate symptoms in some patients, require individualized assessment and careful clinical implementation (4).

In the Asia-Pacific region, an additional challenge is the lack of standardized recommendations, which reflects both an insufficient number of studies and variability in clinical practice (9).

Importantly, the evidence supporting dietary interventions in SIBO remains limited and heterogeneous. Most available studies involve relatively small sample sizes, differ substantially in diagnostic criteria, dietary protocols, and outcome measures, and frequently include patients with overlapping IBS. Consequently, direct comparisons between studies are difficult, and the overall strength of evidence remains insufficient to support strong universal dietary recommendations.

Taken together, the findings suggest that differences observed across the analyzed documents may reflect both variability in the available evidence and differing interpretations of the role of the gut microbiota in the pathogenesis of SIBO. Regional differences may also reflect variability in dietary habits, healthcare systems, access to breath testing, availability of rifaximin, and the extent to which nutritional therapy is integrated into gastroenterological care.

Future research should focus on developing standardized dietary recommendations for SIBO, evaluating the long-term effects of dietary interventions on gut microbiota composition and disease recurrence, and facilitating the development of personalized dietary strategies tailored to patients’ clinical and microbiological characteristics.

### Limitations

This analysis has several significant limitations that should be taken into account when interpreting the results.

First, the available scientific data on dietary therapy for SIBO are limited and heterogeneous. Many of the analyzed publications pertain to populations of patients with irritable bowel syndrome (IBS), in which SIBO is considered one of several possible pathophysiological mechanisms, which may limit the direct generalizability of the results to the population of patients with isolated SIBO (1, 4). This heterogeneity also limits the translation of existing recommendations into routine clinical practice, as clinicians are faced with substantially different dietary approaches and varying levels of evidence supporting their implementation. Furthermore, the limited number of included documents and their heterogeneous methodological nature restrict the ability to draw definitive conclusions regarding true regional differences in clinical practice. Consequently, observed differences should be interpreted as differences between the analyzed documents originating from various regions rather than direct evidence of geographically distinct clinical approaches.

Second, there is a lack of high-quality randomized clinical trials that directly compare the efficacy of different dietary strategies in the treatment of SIBO. Consequently, some of the conclusions are based on indirect data, observational studies, or analyses conducted in other disease entities, which weakens the strength of the evidence.

Additionally, studies evaluating probiotics differed substantially with respect to the microorganisms, strains, dosages, formulations, and treatment protocols used, limiting comparisons across studies and preventing formulation-specific conclusions.

Third, differences between regions of the world may stem not only from differing scientific recommendations, but also from systemic and cultural factors as well as the availability of therapeutic interventions, which were not analyzed in detail in this study.

Fourth, the analysis conducted was qualitative rather than quantitative (e.g., in the form of a meta-analysis), which limits the ability to precisely assess the effects of individual dietary interventions and compare their efficacy.

Finally, the impact of dietary interventions on the gut microbiota was assessed primarily based on indirect studies, which highlights the need for further mechanistic and long-term research aimed at evaluating changes in the composition and function of the microbiome in patients with SIBO.

These limitations underscore the need for future prospective and randomized studies using standardized diagnostic criteria and breath-testing protocols. Further research should evaluate the long-term effects of dietary interventions on symptom control, microbial composition, and disease recurrence, while also investigating dietary strategies independently of antibiotic-related confounding factors.

## 5 Conclusion

Despite increasing interest in nutritional approaches, substantial international variability exists in dietary recommendations for SIBO, and clear global standards for dietary management remain lacking. Antibiotic therapy remains the cornerstone of treatment across most regions, whereas the role of dietary therapy varies considerably.

The low-FODMAP diet appears to be effective for symptom management; however, its impact on SIBO eradication, gut microbiota composition, and long-term clinical outcomes remains uncertain. Similarly, recommendations regarding dietary fiber, probiotics, and prebiotics remain heterogeneous and support an individualized approach based on patient characteristics and clinical presentation.

Current evidence supports dietary therapy primarily as an adjunct to medical treatment rather than a stand-alone therapeutic strategy. An integrated approach combining pharmacological and nutritional management appears to represent the most appropriate strategy for patients with SIBO.

## Data Availability

The original contributions presented in the study are included in the article/Supplementary Material, further inquiries can be directed to the corresponding authors.
